# Occupational Therapy Group Interventions Within the Area of Time Use and Occupational Balance: A Scoping Review

**DOI:** 10.1155/oti/9972405

**Published:** 2026-06-25

**Authors:** Maria Lönn, Katrin Häggström Westberg, Ann-Caroline Holst, Lena-Karin Erlandsson

**Affiliations:** ^1^ Department of Health and Care, School of Health and Welfare, Halmstad University, Halmstad, Sweden, hh.se; ^2^ Psychiatry Halland, Region Halland, Halmstad, Sweden; ^3^ Research and Innovation Centre, Region Halland, Halmstad, Sweden

**Keywords:** investigative techniques, mental illness, recovery, research measures, time management

## Abstract

**Introduction:**

Group interventions are one mode of delivery that supports individuals experiencing challenges related to time management and occupational imbalance in their recovery. However, research on available group interventions, their key components, and how to evaluate their potential effects on time use and occupational balance is scarce.

**Aims/Objective:**

This study is aimed at contributing to the development of evidence‐based occupational therapy interventions by describing research that evaluates group interventions for adults within the scope of time use and occupational balance.

**Materials and Methods:**

A scoping review was conducted using four databases. Records published between 2014 and 2024 were identified, and data from the identified group interventions were extracted and summarized. The outcomes and instruments in the different articles were categorized according to Wilson and Cleary′s conceptual model of patient outcomes.

**Results:**

Thirty‐one articles describing 13 different group interventions were included. The most commonly addressed measures within the time use and occupational balance domain were value or satisfaction measures (for occupations), performance measures (including aspects such as activity level, performance, and competence), and occupational balance measures.

**Conclusions:**

The lack of consensus regarding outcome measures for occupational therapy group interventions highlights the need to clarify and relate key concepts within the domain of time use and occupational balance.

## 1. Introduction

Mental illness is one of the leading causes of ill health in the adult population in Europe [1] and worldwide [2]. Occupational therapy group interventions are one type of intervention used to support recovery among individuals with mental health illnesses [3]. Studies have shown that occupational therapy group interventions can increase individuals′ occupational engagement [4] and accelerate the rehabilitation process [5]. According to a review by Rocamora‐Montenegro et al. [3], different types of occupational therapy group interventions occur in mental health settings. These interventions are most common in the psychosocial group category. They address symptoms, occupational balance, and social and work integration. The focus of attention and setup differ among the available occupational therapy interventions [4, 6, 7]. However, for occupational therapists working with people who have trouble managing their time, balancing their daily activities, finding meaningful activities, or living a sedentary lifestyle, group interventions may be a way to support their recovery in occupational therapy settings [8].

Previous literature reviews targeting occupational therapy interventions for improving occupational balance have slightly different focuses. A recent extensive review addressing interventions aimed at promoting occupational balance for adults included 18 studies reporting occupational balance as a primary or secondary measure [9]. However, this review did not separate individual from group interventions and one‐sidedly included randomized controlled trials, observational studies, and pre–post studies. Lentner et al. included studies targeting occupational balance, although without clarifying how occupational balance was targeted, and focused their review on the effectiveness and quality of assessments rather than clarifying the use of the concept. Thus, despite the frequent use of the concepts of time use and occupational balance in occupational therapy literature [10, 11], group interventions delivered by occupational therapists within the scope of time use and occupational balance are poorly described in the literature. One potential explanation for this phenomenon is the multifaceted nature of the concepts, as well as the extensive variety in the categorization of occupational therapy interventions [4, 6, 7]. The concepts of time use and occupational balance are theoretically intertwined. As Eklund and Gunnarsson posit, a variety of occupations, personal appreciation and satisfaction, and time allocation are all factors to be considered [ [10], p. 2]. As posited by Holmefur et al. [12], time management constitutes a fundamental skill for establishing or maintaining occupational balance. Time use interventions, as described by Kirsh et al., are defined as “a means of addressing the activities in which individuals engage during the 24 h of the day and organizing them in ways that enable well‐being, satisfaction, and a sense of occupational balance” [ [7], p. 132]. This intervention was proposed by Kirsh et al. as one of seven distinct intervention types utilized within the context of occupational therapy mental health settings. A review of the literature was conducted in 2019, which included one individualized time use intervention, Action Over Inertia [13], and two group interventions that were designed to address time use and occupational balance. These group interventions, Redesigning Daily Occupations (ReDO) and Balancing Everyday Life (BEL), are both 16‐week group interventions. ReDO is originally designed for individuals with stress‐related disorders [14, 15], and BEL is designed for individuals with mental illness [16]. A review addressing time use was published several years prior [17]. However, this review exclusively presented one group intervention, the ReDO.

A review of occupational therapy group interventions targeting time use and occupational balance may enhance current understanding of the concepts involved, illuminate variations in populations and settings, and map instruments used to evaluate outcomes of the interventions. In the review by Kirsh et al. [7], a range of outcomes resulting from interventions to improve time use or occupational balance were examined. These outcomes encompassed both those based on occupation and those not based on occupation. For instance, outcomes categorized as occupation‐based encompassed time utilization, occupational balance, and occupational engagement. Conversely, outcomes categorized as nonoccupation‐based encompassed perceived stress and self‐esteem [7]. However, a comprehensive description, including the available instruments applied to evaluate outcomes of interventions, is absent. Enhanced knowledge on evidence‐based group interventions within the scope of time use and occupational balance has the potential to engender an enhanced understanding among occupational therapists, possibly facilitating a more tailored fit of interventions to the needs of individuals.

The objective of this study was to contribute to the development of evidence for occupational therapy interventions. More specifically, the study sought to describe the extant research evaluating occupational therapy group interventions for adults within the scope of time use and occupational balance. Moreover, the objective of the study was to ascertain which instruments and outcome measures are utilized in research evaluating occupational therapy group interventions.

## 2. Materials and Methods

A scoping review was selected to address the comprehensive scope of both concepts across diverse contexts and various study designs [18]. The scoping review was preregistered in the Open Science Framework (OSF) at 10.17605/OSF.IO/W9Q78. The guidelines established in the PRISMA extension for scoping reviews were carefully followed throughout the course of this study [19]. A decision was made to also include articles meeting inclusion criteria from the reference lists (thus a supplement to the PRISMA guidelines). This study adhered to the five stages in performing a scoping review as presented by Arksey and O′Malley [18]:(1) identifying the research question, (2) identifying the relevant studies, (3) study selection, (4) charting the data and collating, and (5) summarizing and reporting the data [18].

### 2.1. Identifying the Research Question

The investigative process was guided by two primary research questions: “What research is available evaluating occupational therapy group interventions within the scope of time use and occupational balance?” and “Which instruments and outcome measures are used in research evaluating occupational therapy group interventions within the scope of time use and occupational balance?”

### 2.2. Identifying Relevant Studies

**Figure 1:**
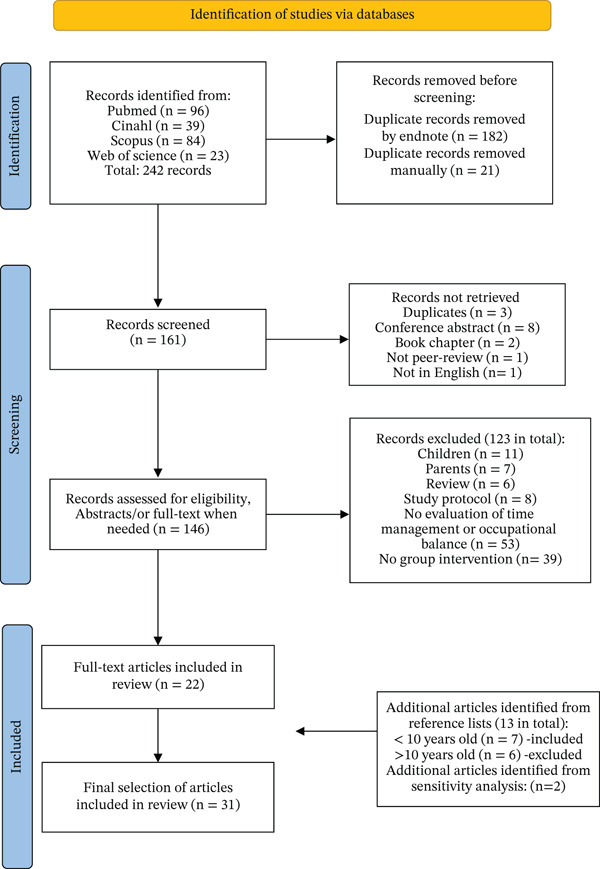
Flow chart (PRISMA).

A systematic search was conducted using the following databases: PubMed, CINAHL, Scopus, and Web of Science. The search was conducted from November 6, 2024, to November 11, 2024. The search was limited in PubMed and Scopus to studies published within the last decade and in English. The search was limited to studies published within the last decade, in English, and in peer‐reviewed journals or journal articles, as queried in CINAHL and Web of Science. The search terms employed in various combinations included time use, time management, occupational balance, a balanced lifestyle, occupational therapy, and group. The final search was conducted on November 11, 2024, and the following search terms were used in PubMed: (“Time Management” [Mesh] OR “occupational balance” OR “everyday balance” OR (“balance” AND “recovery”)). The search terms “occupational therapy” and “group” were also entered into the database. The results from the final search are presented in Figure 1.

### 2.3. Selecting the Studies

The records from the four distinct databases (*n* = 242) were downloaded to EndNote, and duplicates were automatically removed (*n* = 182). Moreover, the first author manually eliminated an additional 21 duplicate records. Subsequently, the records were exported to an Excel spreadsheet, and a review of the records was conducted independently by M.L. and A‐C.H. (*n* = 161). The abstracts were meticulously reviewed, and the articles that met the predetermined inclusion criteria (*n* = 22) were subsequently read in their entirety. In instances where the inclusion criteria remained ambiguous following an initial screening of the abstracts, the articles were thoroughly reviewed in full text. The identification and screening process is delineated in Figure 1.

Inclusion criteria were as follows:

• Adult population
• Group intervention
• An occupational therapy intervention (occupational therapists responsible for the intervention)
• Time use, time management, and/or occupational balance in focus of the intervention

Exclusion criteria were as follows:

• Not published in a peer‐reviewed journal
• Not in English
• Not published in the last 10 years
• Participants of group intervention being children, adolescents, or parents of children
• Conference abstracts
• Study protocol

A total of 47 records were deemed suitable for inclusion, and the results from both reviewers were compared to reach a consensus. A total of 12 records were subjected to a more thorough examination by M.L. and A‐C.H., ending in the determination of exclusion. The rationale behind each exclusion is documented in the flow chart (Figure 1).

A total of 22 articles were identified from various databases and selected for inclusion in the study. A comprehensive review of the reference lists of eligible articles was conducted to identify additional reports (*n* = 7 < 10 years; *n* = 6 > 10 years). Additionally, as a sensitivity analysis, the search term “time use” was added to the initial search in all four databases. This sensitivity analysis resulted in two additional reports that fulfilled the inclusion criteria. This process resulted in the inclusion of nine additional articles (*n* = 7 inclusion of reports from reference lists; *n* = 2 inclusion of reports from sensitivity analysis), bringing the total selection to 31. The inclusion of articles from the reference lists was contingent upon the evaluation of the corresponding group intervention, defined as articles with comparable content and intervention setup. Consequently, other group interventions identified in the reference lists were not incorporated. The ultimate determination of inclusion was arrived at after a deliberative exchange among all four coauthors.

### 2.4. Charting the Data

M.L. and K.H.W. extracted and documented the data from the included articles in an Excel grid. Information on authors, year, design, population, country where the study was performed, aim of the study, outcome measures, and main results was gathered and documented in a systematic manner (see Table 1). Furthermore, a comprehensive array of data was systematically collected for each group intervention, encompassing the objective of the intervention, the number of participants, the duration, the number of sessions, and the categorization of the intervention as manual‐driven or not (see Table 2).

**Table 1:** Description of the identified articles presenting interventions within the domain of time use and occupational balance.

Author, year	Country	Aim of the study	Design of the study and sample size	Setting/population	Outcome measures	Main results
*BEL*						
Argentzell et al., 2020 [20]	Sweden	Investigate if activity engagement, level, and balance mediated and/or moderated change in recovery among persons with mental illness and to explore the possible effect of the BEL intervention compared to standard occupational therapy (SOT).	Cluster RCT. Two hundred twenty‐six participants (133 received BEL and 93 received standard psychiatric treatment).	General psychiatric outpatient care, psychosis teams, and psychiatric community day care settings.	GAF, PMS, **POES**, QPR, and **SDO-OB**	Positive results for recovery improvement with no difference between therapy methods. Activity engagement and mastery strongest mediators of change in recovery.
Eklund, 2023 [21]	Sweden	Evaluate if the BEL intervention was effective compared to standard occupational therapy (SOT) regarding improvement of occupational value and to investigate how self‐mastery, self‐esteem, and sociodemographic factors were related to perceived occupational value.	Cluster RCT. Two hundred twenty‐six participants (133 received BEL and 93 received standard psychiatric treatment).	General psychiatric outpatient care, psychosis teams, and psychiatric community day care settings.	**OVal-pd**, PMS, and Rosenberg Self‐Esteem Scale	Self‐esteem and self‐mastery were positively correlated with all three aspects of occupational value. There were no differences between the two groups regarding change of any of the occupational value outcomes at any of the timepoints.
Eklund and Argentzell, 2023[22]	Sweden	Investigate how occupational therapists and managers viewed the BEL implementation process, with a specific focus on reception and function of the BEL in a multiprofessional team.	Qualitative study with manifest content analysis. Thirteen occupational therapists and three managers.	General specialized psychiatry, psychosis care, community centers, and clinics for depression/anxiety and neuropsychiatric disorders.	Themes	BEL was a welcome alternative, as stated by managers and occupational therapists. Three themes: Conditions and opportunities in the setting, putting the BEL intervention into practice, and experiences of practicing BEL.
Eklund and Bäckström, 2023 [23]	Sweden	Compare two groups receiving either BEL or CAU (care as usual) regarding experiential aspects of readiness for work; to describe change in each of the groups; and to explore sociodemographic and clinical factors, actual work experience, type of intervention, changes in nonwork activity factors, social interaction, and self‐esteem.	Cluster RCT. Two hundred twenty‐six participants (133 received BEL and 93 received standard psychiatric treatment).	General psychiatric outpatient care, psychosis teams, and psychiatric community day care settings.	GAF, ISSI‐SR, **OVal-pd**, **SDO**, Rosenberg Self‐Esteem Scale, and **WRS**	Positive outcomes for both BEL and CAU in work readiness. Satisfaction with nonwork daily activities, self‐esteem, and having valued activities were possible mediators of the treatment effects, whereas intervention type, age, sex, or general activity was not.
Eklund et al., 2023[24]	Sweden	Compare the BEL intervention group with the group receiving standard support regarding motivation for engaging in day care services and outcomes in terms of occupational engagement, personal recovery, and satisfaction with services.	RCT BEL participants 27, standard treatment participants 38.	Day centers.	**POES**, QPR, four items developed for the study, and an amended day care satisfaction scale	The BEL group, but not the standard support group, improved on occupational engagement and recovery. The groups did not differ on any measured aspects of motivation, but motivation for attending the day center was related to service satisfaction.
Eklund et al., 2017 [16]	Sweden	Evaluate the effectiveness of the BEL program for people with mental illness in specialized and community‐based psychiatric services.	Cluster RCT. Two hundred twenty‐six participants (133 received BEL and 93 received standard psychiatric treatment).	General psychiatric outpatient care, psychosis teams, and psychiatric community day care settings.	GAF, MANSA, **OVal-pd**, **POES**, Rosenberg Self‐Esteem Scale, **SDO-OB**, and well‐being assessed by using the first item of the MOS SF‐36	BEL groups improved more in increased activity engagement, activity level, and activity balance after 16 weeks. The group differences on activity engagement and activity level remained at follow‐up, but the CAU group caught up in some areas.
Hultqvist et al., 2019 [25]	Sweden	Explore factors that could predict clinically important improvements in occupational engagement, activity level, occupational balance, and QOL.	Longitudinal study, part of a larger RCT with cluster randomization. One hundred thirty‐three patients from 14 settings.	Outpatient psychiatric clinics, including psychosis teams and psychiatric community day care settings.	GAF, MANSA, PMS, **POES**, Rosenberg Self‐Esteem‐Scale, and **SDO-OB**	BEL showed positive changes in QOL and occupational aspects regardless of sociodemographic details, diagnosis, psychosocial functioning level, severity of symptoms, or the self‐esteem or self‐mastery scores at baseline.
Lund et al., 2019 [26]	Sweden	Explore the processes by which BEL participants made lifestyle changes.	Qualitative study, constructivist grounded theory analysis. Nineteen patients and 29 interviews.	Outpatient psychiatric clinics or community‐based day centers.	Categories	Main results of a process of breaking a cycle of perceived failure and making changes toward a more balanced lifestyle. Five categories: Going at it gently: Change is an ongoing process; support for progress, permission to fail; prioritizing and setting boundaries; adjusting for a sustainable balance; and caring for a valued self.
Lund et al., 2020 [27]	Sweden	Explore group leaders′ and participants′ perspectives of the BEL intervention content and format.	Qualitative study with grounded theory analysis. Twelve group leaders and 19 patients.	Outpatient psychiatric clinics and/or community‐based activity centers.	Categories	Categories were as follows: Appreciating content and structure, desiring flexibility—Both leaders and participants; making connections—BEL as a bridge—Both leaders and participants; and stakeholder‐specific facilitating and hindering factors.
Lund et al., 2019 [28]	Sweden	Explore participants′ perceptions of the group and what brings meaning to the BEL lifestyle intervention.	Qualitative study, grounded theory analysis. Nineteen patients and 26 interviews.	Outpatient psychiatric clinics and/or a community‐based activity center.	Categories	Categories: Joining with others—From feeling alone to connected; a sense of belonging—Mutual support and understanding; and revaluing self‐respect and self‐support.
*LGO*						
Arvidsson et al., 2023 [29]	Sweden	Evaluate the applicability of the intervention (Part 1) by exploring enhancements in time management skills, satisfaction with daily occupations, and aspects of executive functioning.	Intervention study with pre‐ and posttests. Twenty‐one participants.	Habilitation service centers.	**ATMS-S**, **SDO-13**, and WCPA‐SE	Positive change in time management skills and regulation of emotions.
Holmefur et al., 2019 [12]	Sweden	Pilot test of Part 1 of the intervention by exploring enhancements in time management skills, aspects of executive functioning, and satisfaction with daily occupations.	Intervention study with pre‐ and posttests. Fifty‐five participants	Outpatient psychiatric and habilitation settings.	**ATMS-S**, **SDO-13**, and WCPA‐SE	Improvement of time management, organization, and planning skills, emotional regulation, and satisfaction with daily occupations.
Lidström‐Holmqvist et al., 2021 [30]	Sweden	Describe the participant′s experiences and the meaning of attending the intervention (Part 1).	Qualitative design with interviews 1–4 months after the completion of the intervention. Twelve participants.	Outpatient psychiatric clinics and adult habilitation services.	One theme and four categories	Overarching theme: A roller‐coaster process toward control over time in daily life. Four main categories: Understanding why and knowing how, a prerequisite for change; a facilitating learning environment; struggle with implementation in daily life; and perceived outcomes of improved time management.
Wingren et al., 2022 [31]	Sweden	Performed a 1‐year postintervention follow‐up for people with neurodevelopmental or mental disorders.	Single group with a pre–posttest design. Thirty‐eight participants.	Outpatient psychiatric clinics and adult habilitation services.	**ATMS-S**, **SDO-13**, and WCPA‐SE	Improved time management skills, satisfaction with daily occupations, and regulation of emotions after the intervention were maintained after 12 months.
*ReDO*						
Eklund, 2017[32]	Sweden	Follow‐up 3–4 years after intervention on sick leave, well‐being, and occupations.	Extension of a quasiexperimental project. Eighty‐four women (42 who had received the intervention and 42 controls).	Primary healthcare centers.	HADS, **OVal-pd**, PMS, PSS, Rosenberg Self‐Esteem Scale, **SDO-OB**, and percentage of time off sick	Both groups had reduced sick leave. No differences between the groups on well‐being. The intervention group had a better perceived work balance.
Fox et al., 2022 [33]	Ireland	Explore the feasibility of a future RCT and implementation of the ReDo intervention.	Multiphase, mixed‐method design. Ten ReDo participants, nine general practitioners, and two occupational therapists.	Primary healthcare centers. Women with stress‐related health problems and healthcare professionals	DASS, EQ‐5D‐5L, **OVal-pd**, PMS, WHODAS, and four themes and 13 subthemes	Improvements in occupational patterns and all outcomes apart from perceived health. Healthcare professionals were generally positive toward the intervention.
Karlsson et al., 2023 [34]	Sweden	Describe what is needed to achieve a balanced everyday life, including paid work, as experienced by participants of the ReDo intervention.	Qualitative design with content analysis of concluding notes after ReDO intervention from the medical records of 54 individuals.	Primary care setting.	One major theme and four categories	Informants perceived that they must take control of their everyday life as a whole before workability is possible. Major theme: Taking control of one′s everyday life. Categories: Changing the structure of everyday life; social belonging—To have and to handle?; learning to set boundaries; and finding and keeping meaningful occupations.
Olsson et al., 2020[35]	Sweden	Investigate predicted work ability in a long‐term perspective for women at risk for, or on sick leave, after participation in the ReDO‐10 intervention.	Longitudinal cohort study. Eighty‐six women.	Primary healthcare centers.	EQ‐VAS, **OBQ**, **OVal-pd**, PMS, and **WAI**	Improvements in occupational balance, mastery, and occupational value dimensions and perceived health.
*A leisure intervention*						
Farhadian et al., 2024 [36]	Iran	Investigate the feasibility of leisure interventions for individuals with substance use disorder and provide preliminary data for future randomized controlled trials.	Pilot study with pretest, posttest, and 2‐month follow‐up. Nine individuals.	Drug treatment outpatient clinics, local support groups, and Narcotics Anonymous communities. Participants met criteria for substance use disorder and were abstinent for at least 15 days before intervention.	**COPM**, DDQ, NLQ, **OBQ11**, and SF‐36	Improvements in occupational performance, occupational balance, leisure participation, quality of life, and a reduction in drug craving.
*REVEAL(OT)*						
Nielsen et al., 2022 [37]	Denmark	Investigate perspectives of patients and clinicians.	Qualitative study, inductive coding. Three focus group interviews with eight patients and four clinicians.	Physiotherapy, rehabilitation, and anesthesiology departments with a multidisciplinary pain center.	Two themes	Patients reported satisfaction with the intervention and greater acceptance of living with chronic pain, more effective daily planning, and improved social interaction. Two themes: Increased patient acceptance of living with chronic pain and empowering patients to make lifestyle changes.
Nielsen et al., 2022 [38]	Denmark	Evaluate the feasibility and outcomes for adults living with chronic pain.	One‐arm pre–post prospective feasibility study. Thirty‐one adults.	One multidisciplinary pain center and one occupational therapy unit at two hospitals. Patients with chronic pain.	**COPM**, EQ‐5D‐5L, and predefined research progression criteria: Recruitment rate, participant retention, adverse events, program adherence rates, and patient evaluations of relevance, timing, mode of delivery, and procedure acceptance.	Satisfactory program adherence, patients′ self‐perceived relevance, timing and mode of delivery, and assessment procedure acceptance. No improvement in health‐related quality of life but small positive changes in occupational performance and satisfaction.
Nielsen et al., 2023 [39]	Denmark	Explored changes in pain‐related parameters, occupational function and balance, lifestyle factors, and self‐perceived health status in adults with chronic high‐impact pain.	One‐group longitudinal feasibility study. Thirty‐one adults.	Multidisciplinary pain center and patients with chronic nonmalignant pain.	BPI‐sf, CPAQ, EQ‐5D‐5L, KSQ, **OBQ**, PCS, PSEQ, and body pain chart for pain spreading	Lifestyle interventions benefited motor skills, while effects on other outcomes were unclear.
*An occupation-based sleep program*						
Ho and Siu, 2022 [40]	Hong Kong	Investigated the effectiveness of an occupation‐based sleep program for patients with insomnia.	Quasiexperimental design. Forty‐two participants, 22 in the intervention group and 20 in treatment as usual.	Primary care units.	C‐ISI, C‐PSQI, GAD 7, **OB-Quest**, PHQ9, and sleep patterns through an activity wristband	Improvement in sleep duration, occupational balance, and reduction in mood symptoms at posttreatment and 1‐month follow‐up.
*Inpatient energy management education (IEME)*						
Hersche et al., 2019 [41]	Switzerland	Evaluated the feasibility of a research protocol and collected preliminary data on the IEME effect size for patients with multiple sclerosis–related fatigue.	Single‐blinded randomized controlled feasibility study. Twenty‐four participants in IEME and 23 in the control group.	Rehabilitation center.	MMFIS, **OSA**, UW‐SES MS version, SEPECSA, and SF‐36.	The IEME group had significant improvements in self‐efficacy regarding energy conservation strategies.
Hersche et al., 2019 [42]	Switzerland	Developed a group‐based program for people with MS‐related fatigue and completed a pilot program to evaluate OT and participant experiences.	Pilot study with focus group interviews and content analysis. Twelve patients in the intervention, nine of these in the focus group analysis and three OT in another focus group.	Rehabilitation center.		The user experience was positive, and the design feasible within a 3‐week inpatient rehabilitation stay. The IEME was well received, and the topics, supporting materials, and self‐training tasks were useful for the promotion and facilitation of behavioral change.
*An activity-pacing group*						
Søvik et al., 2023 [43]	Norway	To explore participants′ experiences with an activity‐pacing group, how participants perceived self‐managing everyday life after group attendance, and reflections on unmet needs that could enhance self‐management of everyday life with fatigue.	Qualitative study, semistructured interviews with patients with inflammatory rheumatic diseases. Ten participants.	Rehabilitation program at a department of rheumatology.	Themes	Participants increased their understanding of fatigue and their ability to apply strategies to better manage everyday life. Two themes: Acknowledging fatigue and applying self‐management strategies—It is still challenging to manage everyday life, and enhanced management of everyday life requires acknowledgement of fatigue and takes time.
*Everyday Matters: Healthy Habits for University Life digital badge (EMDB)*						
Hunt and Coombes, 2024 [44]	Ireland	Examined the feasibility and acceptability of the intervention, the “Everyday Matters: Healthy Habits for University Life” digital badge, a cocurricular microcredential for first‐year college students.	Single‐arm pre–post design. Eight students completed the demographic questionnaire and pre‐ and postmeasures, with one additional student completing the evaluation/feedback questionnaire only.	First‐year university students.	GQ‐6, **OSA-SF**, RITIS, RU‐SATED, SCS‐SF, and WEMWBS	Improved levels of well‐being, self‐compassion, and growth mindset. Preliminary evidence that the intervention was feasible to deliver and acceptable to first‐year undergraduate students.
*Lifestyle Redesign for Korean university students*						
An and Kim, 2024 [45]	Korea	Investigated effects on Korean university students′ well‐being from aspects of occupational performance, satisfaction, perceived stress level, and QoL.	Quasiexperimental with intervention (*n* = 17) and control group (*n* = 16).	Occupational therapy university students.	**COPM**, **OQ**, SRI, and WHOQOL‐BREF	A statistically significant improvement was observed in occupational performance, and a positive trend in the other areas.
*The Well-Being through Occupational Participation (WBOP)*						
Espiritu and Evetts, 2025 [46]	United States	Assess the efficacy of the intervention in promoting well‐being among occupational therapy graduate students.	Intervention and control group compared with evaluations at three time points—At preintervention, immediate postintervention, and 6 weeks follow‐up. Forty‐one participants: Intervention (*n* = 18) and control (*n* = 23).	Occupational therapy university students.	14‐SGWB, **EMAS**, **OBQ11**, and SCS‐SF	Intervention showed a significant increase in the outcome ratings of general well‐being, self‐compassion, engagement in meaningful occupations, and occupational balance, with effects maintained after a 6‐week period.
*The Pathways to Participation (P2P)*						
Hitch et al., 2022 [47]	Australia	Describe outcomes of group intervention on user identified needs, time use, self‐rated recovery, psychosocial health and community participation.	Descriptive pilot study. Outcomes were measured at baseline, immediately post program and 3 months after program. 17 participants in baseline, 11 contributed post‐program data, and 8 provided follow‐up data.	Adults attending various out‐patient mental health services within a limited geographical area.	CANSAS, RAS‐DS, BASIS‐24, LCQ, time‐use diaries.	Intervention showed reductions in unmet needs and improvements in self‐rated recovery scores but no changes in time use or psychosocial health.
*Action Over Inertia Group Program (AOI-GP)*						
Rees et al., 2022 [48]	Australia	Understand the adapted use of the group‐based intervention from the viewpoints of group participants and facilitators.	Qualitative naturalistic case study. 15 interviews were conducted, 5 group participants twice with 4 weeks apart, 5 group facilitators after completion of group intervention.	Adults with enduring mental illness, and group facilitators from community residential mental health rehabilitation services.	Themes	AOI helped patients identify barriers to more active living, recognize connections between time‐use, health and well‐being, reframe inertia and take steps to overcome it. Facilitators recognized inertia as a major challenge. Two themes: Making Change and Facilitating Change.

*Note:* Outcome measures in bold are defined as outcomes for measurements of time use and occupational balance. Lifestyle Redesign was originally developed in the United States for the elderly as a 9‐month program with individual as well as group sessions ([49]; [50]). The aim and scope of the original program and also other Lifestyle Redesign–inspired interventions found in the reference list of An and Kim (2024) [45] were not included in this review.

Abbreviations: 14SGWB, 14‐Item Scale of General Well‐Being; ATMS‐S, Assessment of Time Management Skills—Short Form; BASIS‐24, The Behavior and Symptom Identification Scale; BPI‐sf, Brief Pain Inventory Short Form; CANSAS, Camberwell Assessment of Need Short Appraisal; C‐ISI, Cantonese Version Insomnia Severity Index; COPM, Canadian Occupational Performance Measure; CPAQ, Chronic Pain Acceptance Questionnaire; CPSQI, Chinese Pittsburgh Sleep Quality Index; DASS, Depression Anxiety Stress Scale; DDQ, Desire to Drug Questionnaire; EMAS, Engaging in Meaningful Activities Survey; EQ5D5L, EuroQol 5‐Dimension 5‐Level questionnaire; EQVAS, EuroQol Visual Analog Scale; GAD7, Generalized Anxiety Disorder 7‐Item Scale; GAF, Global Assessment of Functioning; GQ‐6, The Gratitude Questionnaire‐6; HADS, Hospital Anxiety and Depression Scale; ISI, Insomnia Severity Index; ISSI, Interview Schedule for Social Integration; KSQ, Karolinska Sleep Questionnaire; LCQ, Living in the Community Questionnaire; MANSA,Manchester Short Assessment of Quality of Life; MFIS, Modified Fatigue Impact Scale; NLF, Nottingham Leisure Questionnaire; OBQ, Occupational Balance Questionnaire; OBQ11, Occupational Balance Questionnaire—11 items; OB‐Quest, Occupational Balance–Questionnaire; OQ, Occupational Questionnaire; OSA, Occupational Self‐Assessment; OSA‐SF, Occupational Self‐Assessment—Short Form; Oval‐pd, Occupational Value Assessment—predefined; PCS, Pain Catastrophizing Scale; PHQ9, Patient Health Questionnaire—9‐item scale; PMS, Pearlin Mastery Scale; POES, profiles of Occupational Engagement in people with Severe mental illness; PSEQ, Pain Self‐Efficacy Questionnaire; PSS, Perceived Stress Scale; QPR, Questionnaire about the Process of Recovery; RAS‐DS, The Recovery Assessment Scale—Domains and Stages; RITIS, The Revised Implicit Theories of Intelligence (Self‐Theory) Scale; RU‐SATED, A Self‐Report Instrument for Measuring Sleep Health; SCSSF, Self‐Compassion Scale—Short Form; SDO, Satisfaction with Daily Occupations; SDO‐13, Satisfaction with Daily Occupations‐13; SDO‐OB, Satisfaction with Daily Occupations and Occupational Balance; SEPECSA, Self‐Efficacy for Performing Energy Conservation Strategies Assessment; SF‐36, Medical Outcome Study 36‐Item Short Form Health Survey; SRI, Stress Response Inventory; UW‐SES, University of Washington Self‐Efficacy Scale; WAI, Work Ability Index; WCPA‐SE, Weekly Calendar Planning Activity Swedish version; WEMWBS, Warwick‐Edinburgh Mental Well‐Being Scale; WHODAS, World Health Organization Disability Assessment Schedule 2.0; WHOQOL‐BREF, World Health Organization Quality of Life Scale Abbreviated Version; WRS, Worker Role Self‐assessment.

**Table 2:** The included group interventions (*n* = 13), aims/goals, intervention setup, and indicating if reported as manual‐driven.

Group intervention	Aims and goals of the group interventions	Intervention setup (size of group, no. of meetings, and timeframe)	Manual‐driven intervention
BEL (Eklund et al., [16])	“The BEL program has a strong focus on accomplishing activity balance for the participants, defined as having a satisfying amount of and variation between activities, but also on other aspects of everyday activities, such as activity engagement and valued and satisfying activities. The BEL program also emphasizes personal recovery, which is defined as an individual process toward a meaningful and hopeful life, regardless of the absence or presence of symptoms.”	Five to eight participants per group. The BEL intervention consists of 12 sessions: One session a week for 10 weeks and two booster sessions with 2‐week intervals.	X
LGO (Holmefur et al., [12])	“The goal of LGO is to foster the development of effective time management habits and organizational skills, and it uses cognitive assistive techniques such as maintaining an appointment book and using goal‐directed trial‐and‐error learning strategies for cognitive rehabilitation.”	Six to 12 participants per group. The LGO includes two parts: Part 1 with 10 sessions on daily time management (10 weeks) and Part 2 with six sessions on organization and planning (6 weeks).	X
ReDO [Erlandsson, 15]	“The objective for the ReDO program is that participants, after completing treatment, would have begun a lasting change in patterns of daily occupations and would demonstrate increased work capacity and improved subjective health. The ReDO program is intended to give the participants tools to independently realize the changes they need to make in their patterns of daily occupations.”	Sic to eight participants per group. The program consists of three phases over a total of 16 weeks. Phases I and II are 5 weeks each, and Phase III, job placement, lasts for 6 weeks.	X
A leisure intervention (Farhadian et al., [36])	“The objective of the intervention was to familiarize participants with the concept of leisure, along with identifying both barriers and enablers related to it. The aim was to foster an understanding of preferred and accessible leisure activities, ultimately facilitating the process of leisure planning and engagement.”	Nine participants per group. The program includes two group sessions each week for 2 months.	The intervention and session contents are described in Farhadian et al.′s study (2024). [36] However, no information concerning a manual.
REVEAL(OT) (Nielsen et al., [37])	“The REVEAL(OT) had a three‐fold focus on meaningful activities, healthy eating, and daily physical activity guided by occupational science, occupational lifestyle management research, the World Health Organization′s physical activity guidelines for adults, and the advice on healthy nutrition from the Ministry of Food, Agriculture, and Fisheries in Denmark.”	Six participants per group. The REVEAL(OT) is conducted over 12–15 weeks, including four to eight group sessions of 2 h and two to four individual 1‐h sessions.	X
An occupation‐based sleep program (Ho & Siu, [40])	“An occupation‐based sleep program to promote awareness of sleep hygiene factors, promote an environment conducive to sleep, and restructure participation in daytime activities with a focus on occupational balance.”	Four to six participants per group. Group sessions and individual sessions: 2 h once a week for 8 weeks. The program is described as standardized and includes teaching materials.	No information concerning a manual.
Inpatient energy management education (IEME) (Hersche et al., [51])	“The goal of the IEME is to ensure that participants learn how to manage available energy in order to achieve a satisfying and meaningful daily routine.”	Two to seven participants per group. Individual session before and after the five group sessions. In total, 5 h (each session 1 h) group sessions for 3 weeks.	X
An activity‐pacing group (Søvik et al., [43])	“The intervention aims to enhance participants′ motivation for change and enable them to better manage everyday life. Through individual reflection, tasks, and group discussions, the intervention places an emphasis on increasing participants′ awareness of perceived challenges and how they can self‐manage.”	The number of participants in each group is not defined. An activity‐pacing group consisting of two 1‐h group sessions as part of a larger rehabilitation program (conducted during 2–4 weeks). An activity log and activity plan are completed individually before the first group session.	The intervention and session contents are described in Søvik et al.′s study (2023). [43] However, no information concerning a manual.
Everyday Matters: Healthy Habits for University Life digital badge (EMDB) (Hunt & Coombes, [44])	“With the goals of supporting first‐year college students, increasing their preparedness for college life and their chances of study success, participants in the EMDB are encouraged to explore some practical everyday things that they can say and do to support themselves as much as possible during this time of change and establish healthy habits and routines for their university lifestyle, enhancing their successful transition into and through higher education.”	Attendance in group sessions ranged from 10 to 14 (max 30 participants). One‐hour lunch sessions in groups for 8 weeks. The intervention content is described briefly in Søvik et al.′s study [43].	No information concerning a manual.
Lifestyle Redesign^a^ for Korean university students (An & Kim, [45])	“The primary focus of this intervention has centered on effectively managing and planning the utilization of time and activity patterns, promoting the adoption of healthy habits, and encouraging meaningful activity engagement, all aimed at enhancing the overall quality of life for the participants.”	Three to four participants per group. Thirty‐minute individual sessions once each week. One‐hour group sessions once per week for 10 weeks. The intervention and session contents are described in Hunt and Coombes′ study [44].	No information concerning a manual.
The Well‐Being through Occupational Participation (WBOP) (Espiritu et al., [46])	“The WBOP intervention was based on the theory of occupational adaptation with the goal of increasing the internal adaptation process and facilitating an individual′s ability to overcome occupational challenges.”	Eighteen participants in the intervention group (min–max not defined). Forty‐five‐minute virtual group sessions once per week for 6 weeks. The intervention content is described in An and Kim′s study [45].	No information concerning a manual.
The Pathways to Participation (P2P) Program (Hitch et al., [47])	“The P2P intervention is a hybridized program that aims to enable consumers to engage in meaningful activities at all stages of recovery. The P2P program combines two evidence‐based interventions, the Action Over Inertia (AOI) program and the WORKS program. The program emphasizes gradual momentum toward personal recovery goals and aims to increase engagement, enhance activity balance, promote social interaction and community engagement, and improve overall health and well‐being.”	Seventeen participants were recruited to the intervention (participants per group not defined). Two‐hour sessions once each week for 4 + 6 weeks. The content during the first 4 weeks is based on the AOI program, and the content during the next 6 weeks is based on the WORKS program. The intervention and session contents are described in Hitch et al.′s study [47].	X
Action Over Inertia Group Program (AOI‐GP) (Rees et al., [48])	“Action Over Inertia (AOI) is a flexible workbook‐based, time use intervention for use in collaboration with people experiencing challenges of everyday living with severe mental illness. In Australian community mental health settings, occupational therapists have begun using AOI in a group format to not only promote understanding of the contribution of activity participation in recovery and well‐being but also to foster group support for self‐development and effecting change.”	Five participants in the intervention group (min–max not defined). An individualized program, at varying pace and length, is run over five to eight sessions.	A locally developed AOI facilitator guide.

^a^Lifestyle Redesign was originally developed in the United States for elderly individuals as a 9‐month program comprising individual and group sessions ( [49]; [50]). The objective and breadth of the original program and other Lifestyle Redesign–inspired interventions identified in the reference list of An & Kim [45] were not encompassed in this review. Despite drawing from the same program, the setup and themes differed markedly; thus, Lifestyle Redesign for university students is presented as a discrete intervention, and the articles from the reference list are not included in this review.

### 2.5. Collating, Summarizing, and Reporting the Data

The authors carefully reviewed existing research on occupational therapy group interventions, focusing on their time‐based aspects and the promotion of occupational balance. The instruments utilized for the evaluation of outcomes were classified in accordance with the conceptual model of patient outcomes proposed by Wilson and Cleary [52]. A strength of this model is the primary focus on conceptualizing patient outcomes, which suited the aim of the present study. The categorization was executed in a deductive manner, and instruments were marked and labeled if assessed to measure any aspect of time use or occupational balance. The instruments were then categorized according to the aspects measured by individual variables (such as included subscales in specific instruments). Consequently, an instrument was classified into a category if the included subscale (outcome measure) was evaluated as being associated with that category (this was conducted irrespective of the manner in which the specific instrument and included subscales were utilized in the included studies). The categorization of outcome measures was the subject of discussion among the coauthors until a consensus was reached.

## 3. Results

### 3.1. General Description of the Included Studies

A total of 31 articles published between 2014 and 2024 satisfied the established inclusion criteria. The studies included are presented in Table 1. A total of 13 distinct group interventions were identified among the included studies (Table 2). The most frequently described intervention was BEL, with 10 studies, followed by Let us Get Organized (LGO) and ReDO (each with four studies). Redesign Your Everyday Activities and Lifestyle with Occupational Therapy (REVEAL(OT)) was described in four studies, and the inpatient energy management education (IEME) program was described in two studies. The remaining eight group interventions only appeared in one study each. The group interventions included in this review were developed in Sweden (*n* = 3), Denmark (*n* = 1), Iran (*n* = 1), Hong Kong (*n* = 1), Switzerland (*n* = 1), Norway (*n* = 1), the United States (*n* = 1), Ireland (*n* = 1), Australia (*n* = 2), and Korea (*n* = 1). The Scandinavian countries constituted the settings that were presented most frequently. The settings and targeted populations identified in the included occupational therapy interventions exhibited significant heterogeneity, encompassing a wide spectrum of symptoms, diagnoses, and issues. The most frequently observed settings included outpatient settings such as general psychiatric care, psychosis teams, and community centers/services (10 studies evaluating BEL, one study evaluating the Action Over Inertia Group Program (AOI‐GP), and one study evaluating the Pathways to Participation (P2P) Program); drug treatment outpatient clinics (one study evaluating a leisure intervention); and outpatient psychiatric clinics and habilitation centers (four studies evaluating LGO). A smaller number of studies focused on alternative rehabilitation centers, with a total of six studies assessing the group interventions REVEAL(OT), the IEME program, and an activity‐pacing group. A similar trend was observed in primary healthcare settings, with five studies in total, four of which evaluated ReDO and one study examining an occupation‐based sleep program. Finally, three studies in university settings assessed the Well‐Being through Occupational Participation (WBOP), Everyday Matters: Healthy Habits for University Life digital badge (EMDB), and Lifestyle Redesign.

The group interventions that were the subject of this scoping review were characterized by group sizes ranging from two to 30 individuals (Table 2). However, the most common group size was four to eight individuals. The intervention time frames ranged from 2 to 16 weeks (see Table 2). The objectives and goals of the various group interventions are enumerated in Table 2. All group interventions were designed with the objective of promoting change, as evidenced by specific language such as “accomplishing,” “process,” “strategies,” “change management,” “facilitating,” “restructuring,” “enabling,” “adaptation,” “support,” “establishing,” and “enhancing.” The following challenges were addressed: time management habits and organizational skills, daily occupation and activity patterns, daily routines, healthy habits, lifestyle management, participation, engagement, and management in daytime activities and everyday life, satisfying and meaningful activities, activity balance and occupational balance, energy management, and occupational challenges.

Of the 13 interventions, six were presented as manual‐driven, and one included a facilitator guide (Table 2). Two of the interventions were described in detail in a publication; however, no available information was found regarding whether they were manual‐driven. Consequently, four of the publications concerning interventions did not encompass the content of the sessions or the specifics of delivery.

### 3.2. Outcome Measures Used in the Included Studies

**Figure 2:**
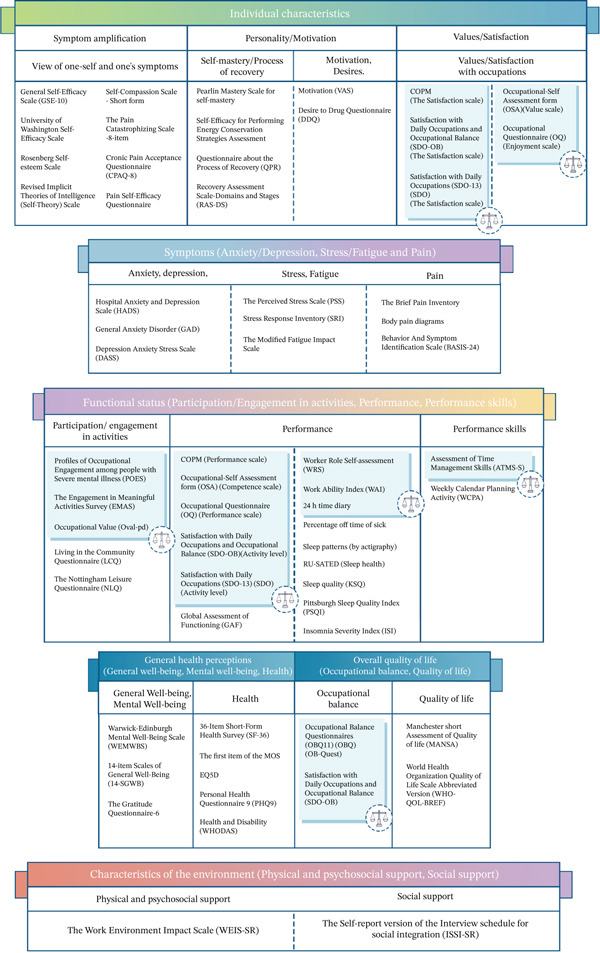
Conceptual map of outcome measures/instruments used in research evaluating group interventions within the scope of time use and occupational balance. The instruments were categorized according to individual characteristics, symptoms, functional status, general health perceptions, overall quality of life, and characteristics of the environment. Some of the outcome measures applied in the included studies were multi‐item scales, that is, several items were used to measure specific constructs. The categorization presented is based on the constructs they measured, and in some cases, constructs from subscales are presented (in brackets). The scale symbol is used to illustrate instruments categorized as measuring aspects of time use/occupational balance.

The outcome measures were categorized according to the following criteria: individual characteristics (*n* = 19), symptoms (*n* = 9), function (*n* = 23), health (*n* = 8), quality of life (*n* = 6), and environment (*n* = 2) (as described by Wilson and Cleary [52]). A complete description of the outcome measures included can be found in Table 3, with associated references outlined in Supporting Information 1. Additionally, outcome measures were assessed for any aspect of time use or occupational balance, utilizing a total of 15 unique instruments/assessment tools (see Figure 2 and Table 1).

**Table 3:** Outcome measures applied in the included studies.

	BEL	LGO	ReDO	A leisure intervention	REVEAL(OT)	An occupation‐based sleep program	IEME	An activity‐pacing group (no measures reported)	EMDB	Lifestyle Redesign	WBOP	P2P	AOI‐GP
*Individual characteristics (symptom amplification, personality/motivation, and values/satisfaction)*													
General Self‐Efficacy Scale (GSE‐10) (Schwarzer et al., [53])		X											
University of Washington Self‐Efficacy Scale (Amtmann et al., [54])							X						
Rosenberg Self‐Esteem Scale (Rosenberg, [55])	X		X										
Revised Implicit Theories of Intelligence (Self‐Theory) Scale (De Castella et al., [56])									X				
Self‐Compassion Scale—Short Form (Raes et al., [57])									X		X		
Pain Catastrophizing Scale (Sullivan et al., [58] Osman et al., 1997 [59])					X								
Chronic Pain Acceptance Questionnaire (Eide et al., [60] Rovner et al., [61] Fish et al., [62])					X								
Pain self‐efficacy (Rasmussen et al., [63])					X								
Pearlin Mastery Scale for self‐mastery (Pearlin et al., [64])	X		X										
Self‐Efficacy for Performing Energy Conservation Strategies Assessment (Liepold et al., [65])							X						
Questionnaire about the Process of Recovery (QPR) [Argentzell et al., 66]	X												
Recovery Assessment Scale—Domains and Stages (RAS‐DS) (Hancock et al., [67] Scanland et al., [68])												X	
Motivation (VAS) [Eklund & Tjornstrand 69]	X												
Desire to Drug Questionnaire (DDQ) (Hassani‐Abharian et al., [70])				X									
Canadian Occupational Performance Measure (satisfaction scale) (COPM) [Law et al., 71]				X						X			
Satisfaction with Daily Occupations and Occupational Balance (SDO‐OB) (satisfaction scale) [Eklund & Argentzell 72]	X	X	X										
Satisfaction with Daily Occupations (satisfaction scale) (SDO‐13) (SDO) [Eklund et al., 73]	X	X											
Occupational Self‐Assessment—Short Form (OSA‐SF) [Popova et al., 74, Keilhofner et al.,^75^]									X				
Occupational Self‐Assessment (value scale) (OSA) (Keilhofner et al., [75])							X						
Occupational Questionnaire (enjoyment scale) [Smith et al., 76]										X			
*Symptoms (anxiety/depression, stress/fatigue, and pain)*													
Hospital Anxiety and Depression Scale (HADS) (Zigmond and Snaith, [77])			X										
General anxiety disorder (GAD) (Spitzer et al., [78])						X							
Depression Anxiety Stress Scale (DASS) (Lovibond et al., [79])			X										
The Perceived Stress Scale (PSS) (Cohen et al., [80])			X										
Stress Response Inventory (SRI) (Koh et al., [81])										X			
The Modified Fatigue Impact Scale (Kos et al., [82])							X						
The Brief Pain Inventory (Cleeland et al., [83])					X								
Body pain diagrams (Southerest et al., [84])					X								
Behavior and Symptom Identification Scale (BASIS‐24) (Cameron et al., [85])												X	
*Functional status (participation/engagement in activities, performance, and performance skills)*													
Profiles of Occupational Engagement among people with Severe mental illness (POES) [Bejerholm et al., 86; Bejerholm and Lundgren‐Nilsson, 87])	X												
The Engagement in Meaningful Activities Survey (EMAS) [Eakman, 88, Goldberg et al., 89])											X		
Occupational value (Oval‐pd) [Eklund et al., 90], [Eklund et al., 91]	X		X										
Living in the Community Questionnaire (LCQ) (Australian Health Ministers Advisory Council, [92])												X	
Nottingham Leisure Questionnaire (NLQ) (Drummond et al., [93] Altintas et al., [94])				X									
Canadian Occupational Performance Measure (performance scale) (COPM) [Law et al., 71]				X						X			
Occupational Self‐Assessment (competence scale) (OSA) (Keilhofner et al., [75])							X						
Occupational Questionnaire (performance scale) [Smith et al., 76]										X			
Satisfaction with Daily Occupations and Occupational Balance (SDO‐OB) (activity level) [Eklund & Argentzell, 72]	X	X	X										
Satisfaction with Daily Occupations (SDO‐13) (activity level) (SDO) [Eklund et al., 73]	X	X											
Camberwell Assessment of Need Short Appraisal (CANSAS) (Slade et al., [95] Trauer et al., [96])												X	
Global Assessment of Functioning (GAF) (Endicott et al., [97])	X												
Worker Role Self‐Assessment (WRS) [Eklund & Bäckström, 91]	X												
Work Ability Index (WAI) (Ahlström et al., [98])			X										
24 h time diary												X	
Percentage of time off sick			X										
Sleep patterns (by actigraphy)						X							
RU‐SATED Questionnaire (Ravyts) [99]									X				
Sleep quality (KSQ) (Nordin et al., [100])					X								
Pittsburgh Sleep Quality Index (PSQI) (Backhaus et al., [101])						X							
Insomnia Severity Index (ISI) (Bastien et al., [102])						X							
Assessment of Time Management Skills (ATMS‐S) [Janeslätt et al., 103]		X											
Weekly Calendar Planning Activity (WCPA) [Toglia, 104]		X											
*General health perceptions (general well-being, mental well-being, and health)*													
Warwick‐Edinburgh Mental Well‐Being Scale (WEMWBS) (Tennant et al., [105])									X				
14‐Item Scales of General Well‐Being (14‐SGWB) (Longo et al., [106])											X		
The Gratitude Questionnaire‐6 (Mccullough et al., [107])									X				
36‐Item Short‐Form Health Survey (SF‐36) (Ware and Sherbourne, [108])				X			X						
The first item of the MOS SF‐36 (Bowling, [109])	X												
EQ5D (Devlin et al., [110] Janssen et al., [111] Herdman et al., [112])			X		X								
Personal Health Questionnaire 9 (PHQ9) (Löwe et al., [113])						X							
Health and disability (WHODAS) [Üstün et al., 114]			X										
*Overall quality of life (occupational balance and quality of life)*													
Occupational balance (OBQ11) [Håkansson et al., 115]				X							X		
Occupational balance (OBQ) [Wagman & Håkansson, 116]			X		X								
Occupational balance (OB‐Quest) (Dur et al., [117])						X							
Satisfaction with Daily Occupations and Occupational Balance (occupational balance scale) (SDO‐OB) [Eklund & Argentzell, 72]	X	X	X										
Manchester Short Assessment of Quality of Life (MANSA) (Priebe et al., [118])	X												
World Health Organization Quality of Life Scale Abbreviated Version (WHOQOL‐BREF) (Min et al., [119])										X			
*Characteristics of the environment (physical and psychosocial support and social support)*													
The Work Environment Impact Scale—Self‐Rating (WEIS‐SR) (Wastberg et al., [120])			X										
The self‐report version of the Interview Schedule for Social Integration (ISSI‐SR) (Unden et al., [121] Eklund et al., [122])	X												

Outcome measures targeting individual characteristics were prevalent and classified according to the three domains delineated by Wilson and Cleary [52]: symptom amplification, personality/motivation, and values/satisfaction. Among the instruments that were included in the study, the satisfaction scales (in SDO‐OB, SDO/SDO‐13, and COPM) and measurements of value (including the OSA/OSA‐SF and OQ) were considered aspects targeting time use or occupational balance. The SDO‐OB [72] was utilized in the evaluation of BEL, LGO, and ReDO, while the SDO/SDO‐13 [73] was employed in the BEL and LGO evaluations. The COPM [71] was employed in a leisure intervention and Lifestyle Redesign study, and the OSA/OSA‐SF [74, 75] was utilized in the IEME and EMDB studies. The OQ [76] was utilized exclusively in the Lifestyle Redesign study (*n* = 1) (Table 3).

Outcome measures targeting function were also commonplace. A categorization was performed according to three categories deemed appropriate by the research team: participation/engagement in activities, performance, and performance skills. As illustrated in Figure 2, all previously mentioned categories were found to be pertinent when assessing time utilization and occupational balance. Aspects of performance or activity level were incorporated into the following instruments: SDO‐OB, SDO/SDO‐13, COPM, OSA/OSA‐SF, and OQ. These instruments have previously been mentioned and thus include measurements of satisfaction/value as well as measurements of performance/activity level (see Figure 2). Instruments specifically targeting and acknowledging the aspect of time/extent of time in different activities included the POES [86] and a time use diary.

Outcome measures targeting health or quality of life were utilized in the evaluation of all group interventions, except for the activity‐pacing group, for which no outcome measures were incorporated. The research team assessed outcome measures targeting occupational balance as part of an individual′s perception of quality of life. The following instruments were included in the assessment: OBQ/OBQ11 [115, 116], OB‐Quest [117], and SDO‐OB [72]. The OBQ/OBQ11 was utilized in ReDO, REVEAL(OT), a leisure intervention, WBOP, OB‐Quest in an occupation‐based sleep program, and the SDO‐OB in BEL, LGO, and ReDO (Table 3).

The LGO was the sole group intervention that incorporated instruments for assessing performance skills, such as the ATMS [123] and WCPA [104] (see Figure 2).

## 4. Discussion

The present scoping review sought to contribute to the development of evidence for occupational therapy interventions, specifically by describing available recent research evaluating occupational therapy group interventions within the scope of time use and occupational balance.

A total of 31 articles were identified, which presented 13 distinct group interventions addressing the domains of time use and/or occupational balance. The group interventions addressed a broad range of occupational challenges, with a focus on supporting individuals to develop healthier daily activity patterns and routines, enhance engagement in meaningful activities, achieve occupational balance, and manage energy, time, and everyday occupational demands. A total of 61 instruments were utilized in the 13 group interventions that were included in the study. A total of 15 instruments/assessment tools relevant to time use and occupational balance were assessed based on their relevance to individual characteristics, functional status, and quality of life. The paucity of predefined primary outcome measures, the expected magnitude of change, and, furthermore, a complex variety of measures are indicative of a fundamental limitation in the design of occupational therapy research.

The diversity of the 13 group interventions identified in this review can be seen as a reflection of the richness of occupational therapy practice across different contexts. However, the predominance of interventions developed in Scandinavian countries, particularly Sweden, may be discussed in relation to cultural transferability. The way interventions are structured, delivered, and received is likely influenced by a variety of factors, including cultural norms, societal expectations, and particularly differences in healthcare systems. The existence of an international consensus regarding the concepts of occupational balance or time use remains unconfirmed, and these concepts may be interpreted differently depending on the cultural context. Consequently, further research is necessary to investigate the performance of these interventions when implemented in countries other than their country of origin. It is imperative to ascertain whether cultural adaptation is necessary to ensure relevance and effectiveness.

A mere six of the 13 interventions were characterized as manual‐driven, and numerous publications were found to be lacking in terms of providing detailed information regarding session content or delivery methods. This dearth of transparency imposes limitations on the capacity to replicate and systematically evaluate interventions. A manual for sessions, which includes content and methods, not only supports fidelity in implementation but also facilitates training, scalability, and comparison across studies. In the context of evidence‐based practice, it is imperative to have a clearly defined and documented intervention protocol to assess outcomes and ensure consistency. The paucity of documentation in several of the studies included in the present review is consistent with the conclusions of Birken et al. [124], underscoring the significance of rigorous reporting in scientific studies for deriving robust conclusions. Consequently, there is a necessity for enhanced reporting standards and greater emphasis on intervention development processes in occupational therapy research.

In this review, occupational balance was categorized as a component of an individual′s quality of life. The association between occupational balance and quality of life has been demonstrated in previous research [125–127]. However, there is a lack of consensus on the dimensions and outcome measures of well‐being and quality of life. To address this issue, frameworks have been proposed to be context‐specific for improved precision and replication in mental health research [128]. In the 2017 study, Eklund et al. sought to delineate the notion of occupational balance. This endeavor yielded three overarching themes encompassing outcome measures employed as indicators of occupational balance. The first theme pertains to a harmonic mix of occupations or activities. The second theme concerns the ability and resources necessary for engagement in occupations. The third theme focuses on the congruence between an individual′s occupational engagement and their values and personal meaning [129].

The initial theme in Eklund et al. [129] is a harmonic mix of occupations, which refers to the optimal variation between different occupations for each individual. This theme is suggested to be measured with instruments such as the OBQ, OB‐Quest, and SDO‐OB [129]. This finding is consistent with the categorization in the current review, in which OBQ, OB‐Quest, and SDO‐OB were also classified as instruments measuring occupational balance. Eklund et al. [129] initiated an exploration of the thematic linkages of occupational balance and time use, their interrelationships, and underlying aspects; however, this was not examined in depth. In the present review, the instruments COPM, SDO‐OB, SDO‐13, SDO, OQ, OSA‐SF, and Oval‐pd were divided and categorized as measures of time use and/or occupational balance. These instruments were assessed as belonging to both “individual characteristics” and “functional status.” This classification was guided by the distinction between functional performance and subjective evaluation within the Wilson and Cleary model. The included instruments assess both participation in occupations and satisfaction with the ability to perform those occupations. Satisfaction was interpreted as a subjective evaluation, aligned with higher level constructs such as general health perceptions and quality of life. This interpretation is supported by previous literature suggesting that satisfaction with daily occupations reflects perceived well‐being and meaning rather than performance alone [11, 88]. Accordingly, the instruments were classified across two domains based on the aspects measured within their components rather than as whole measures.

Weighing these two aspects could thus reflect the third theme as described by Eklund et al.: “…a match between actual and desired activity configuration” [129] p. 51. However, in the instruments included in this review, the measurement of such weighting was often not addressed, and it was not intended as an outcome, nor, as far as we know, not addressed. In addition, the question of whether an increase in perceived well‐being and meaning is a consequence of participation in an occupational therapy group intervention remains an area that requires further investigation.

The theme ability and resources needed to engage in occupations, as described by Eklund et al. [129], were also commonly addressed in the present scoping review. The impact of these factors was evident in individual characteristics and outcome measures, such as self‐efficacy and self‐mastery. However, the relationship between these factors and specific ongoing occupations was not always clear. In this review, individual characteristics were assessed in relation to three distinct domains: an individual′s value and satisfaction, particularly in connection to occupational engagement (e.g., satisfaction and value in one′s performance of occupation), personality and motivation (e.g., self‐mastery, recovery processes, and motivation), and symptom amplification (e.g., self‐efficacy, self‐esteem, and pain acceptance). A more thorough examination is necessary to elucidate the relationship between the concepts in this review that pertain to individual characteristics and their correlation with occupational balance.

Our results indicate that performance skills were seldom utilized as an outcome measure in studies of occupational therapy group interventions. One exception was time management skills and measures of executive function (used as outcome measures in LGO). Time management skills are defined as the ability to “order events in chronological sequence and allocate amounts of time into events and activities” [123]. The ATMS‐S is a self‐reported questionnaire concerning time management skills [123]. The WCPA has been identified as a reliable measure of executive function in “ecological everyday situations,” which encompasses a range of skills including time management, organization, and planning [30, 104]. The utilization of both instruments was exclusively for the purpose of evaluating LGO. Executive functions are required to manage multiple tasks in everyday life, including higher level cognitive skills necessary for selecting, initiating, implementing, and overseeing functional behavior and expression of emotions [130]. However, the selection of outcome measures should be informed by the objective of the intervention, whether to enhance executive functions or to assess compensatory skills. Outcome measures that are sensitive to, and, furthermore, expected to change, are of greatest importance. A more robust rationale, substantiated by theory, has the potential to enhance future research and assist researchers in selecting appropriate outcome measures.

The utilization of disparate outcome measures and the selection of instruments are insights that, to the best of our knowledge, have been underappreciated in the context of occupational therapy interventions. The findings outlined in this study have the potential to inform occupational therapists working in clinical settings. Furthermore, knowledge gained can be used to promote collaboration among occupational therapists within the research domain, thereby in the long term improving the comparability of research findings and contributing to the strengthening of evidence‐based occupational therapy practices.

The primary outcome generally serves as the primary metric for evaluating the efficacy of the intervention. Consequently, other outcomes are considered secondary outcomes [131]. The selection of outcome measures is of paramount importance. For instance, Treweek et al. [132] concluded in their examination of late‐stage cancer treatment trials that there was a 28% agreement among a group of patients and healthcare professionals with experience of this type of study regarding which primary outcome should be used. In essence, the outcome measure has the potential to bolster the evidence of effectiveness; however, there is a concomitant risk that the measure may not be pertinent or may even fail to demonstrate the alterations precipitated by the intervention. The initial step in this direction is to establish clarity regarding the knowledge that can be acquired from an intervention study [131]. In addition, the utilization of a framework such as the International Classification of Functioning, Disability and Health (ICF) or the Wilson and Cleary conceptual model, as employed in this review, is recommended to identify pertinent outcomes [131].

The results of this study indicate significant variation in the choice of outcome measures. Most of the studies included did not specify or describe the methodology for measuring a primary outcome. The scope of this study cannot verify whether this phenomenon is a limitation in the design of the specific studies included in the analysis or a general reflection of occupational therapy research. However, we believe that future research and evaluation of occupational therapy interventions should include a clear account of the aim, hypothesis, expected change, and primary outcome measure.

### 4.1. Methodological Considerations

The reliability of this scoping review was strengthened by following the five‐step framework proposed by Arksey and O′Malley [18]. Each stage of the process was systematically documented and discussed among the coauthors, and no stage was conducted in isolation. This collaborative approach ensured transparency and reduced the risk of individual bias. However, a methodological limitation concerns the search strategy. A sensitivity analysis was conducted to compensate for this limitation, resulting in the inclusion of two additional records. A more extensive array of search terms and an extended time frame might have led to the identification of additional relevant records. For example, additional search variations of time use and time management are suggested for future reviews. The present strategy entails potential for selection bias, a factor that must be considered when assessing the findings′ scope and representativeness.

As previously indicated, most of the interventions encompassed in this review were developed in Scandinavian countries (n = 5), notably Sweden. While studies from outside Europe have emerged more recently (e.g., Australia, Iran, Hong Kong, and Korea), the overall picture remains consistent with previous analyses of occupational therapy research, which have highlighted its concentration in economically privileged, English‐speaking, or Western countries [8, 133, 134]. This distribution, characterized by its unevenness, has the potential to restrict the generalizability of the findings. It is important to note that this phenomenon is indicative of more extensive structural challenges that pervade the realm of occupational therapy research. These challenges include, but are not limited to, underfunding, a paucity of methodological diversity, and publication barriers that are particularly pronounced in non‐English contexts.

The review′s primary focus was group interventions related to time use and occupational balance. However, interventions classified as lifestyle‐focused or habit‐changing [7] exhibit conceptual overlaps with several of the included programs, including Lifestyle Redesign. This overlap gives rise to challenges in terms of categorization and synthesis. Furthermore, the adaptation of intervention manuals to local contexts or populations introduces additional layers of complexity, which further complicates the evaluation process. For instance, the Lifestyle Redesign for Korean university students was regarded as a distinct intervention in this review, while the original Lifestyle Redesign [49, 50], developed in the United States, was not identified. Such variations have the potential to introduce bias in terms of the elements that are captured and excluded.

To classify the instruments used in the reviewed studies, the Wilson and Cleary model [52] was applied. This model conceptualizes outcomes along a continuum from physiological factors to overall quality of life and has been widely used to examine relationships between clinical variables and perceived health (e.g., [135], [136]). The concepts of occupational balance and time use are both considered multidimensional constructs, involving not only the distribution and variety of occupations but also subjective experiences such as meaning, satisfaction, and perceived balance [11, 88, 129]. The included outcome measures were therefore expected to span several domains. The Wilson and Cleary model enabled the inclusion and organization of a broad range of instruments across hierarchical levels. However, the model does not explicitly incorporate occupational therapy–specific constructs. An alternative approach could have been to apply the ICF [137], which is commonly used in occupational therapy research and provides a comprehensive framework for functioning and contextual factors. Nevertheless, the ICF was considered to be less suited for categorizing multidimensional outcome measures that include subjective well‐being.

Even if aspects related to “ability to do” and improving occupational participation are expected primary outcomes of occupational therapy interventions, evaluations of occupational therapy interventions cannot be expected to rely solely on occupation‐focused outcome measures. In practice, occupational therapy interventions are frequently assessed using a combination of symptom‐based, functional, and quality of life measures. Consequently, a classification approach limited to occupation‐focused constructs may risk excluding relevant outcomes. A broader framework that captures multiple dimensions of health was therefore considered more appropriate for the present review. In this context, Wilson and Cleary′s model made it possible to include and organize various outcome measures at different hierarchical levels while still considering both functional and subjective aspects relevant to occupational therapy.

Notwithstanding the study′s limitations, this review contributes to the expanding evidence base for occupational therapy group interventions. An earlier review concluded that research in this area was too scarce to allow a systematic evaluation [7]. By including a broader range of group interventions that extend beyond mental health, this review highlights the potential of such approaches and emphasizes the need for clarity in the selection of outcome measures to strengthen the evidence base.

## 5. Implications for Occupational Therapy

• The article provides a comprehensive overview of the various types of occupational therapy group interventions available, with a focus on the evaluation of their impact on time utilization and occupational balance.
• The group interventions that have been included in this study reflect the diversity of occupational therapy practice. However, with most studies originating from Scandinavia, questions are raised about the cultural transferability of these interventions. The emergence of common themes may pinpoint the main core and facilitate adaptation according to cultural differences, aspects that may be considered in the presentation and documentation of occupational therapy group interventions.
• The dearth of comprehensive and adequate documentation of group interventions may hinder the replication, evaluation, and broader implementation of said interventions.
• The variation in identified outcome measures underscores the need for clear occupation‐based outcome measures, together with clarification of domains under evaluation, which would allow for comparability across different studies to support the development of evidence‐based occupational therapy.
• Future research may evaluate the correlation between different outcome measures and increase the knowledge base concerning associations between outcome measures targeting time use and occupational balance.

## 6. Conclusion

The present review identified 13 group interventions targeting time use and occupational balance. The interventions addressed a range of perspectives, including the development of healthier daily activity patterns and routines, the enhancement of engagement in meaningful activities, and the management of energy, time, and everyday occupational demands. The group interventions that were the focus of this study incorporated various instruments that addressed components related to the utilization of time, as reflected in performance, participation, or engagement in occupations. Additionally, the interventions encompassed dimensions that reflected personal meanings in and satisfaction with occupational engagement. This partition can be used to deepen our understanding of the concept of occupational balance. The wide range of outcome measures, combined with the lack of consensus regarding the design and outcome, underscores the need for further studies and more precise, occupation‐focused guidelines to strengthen the field of evidence‐based occupational therapy.

## Author Contributions

Study concept and design: Maria Lönn, Katrin Häggström Westberg, and Lena‐Karin Erlandsson. Data collection, screening, and extraction: Maria Lönn, Katrin Häggström Westberg, and Ann‐Caroline Holst. Data analysis and synthesis: Maria Lönn, Katrin Häggström Westberg, and Lena‐Karin Erlandsson. Drafting of the manuscript: Maria Lönn. Revision and final approval: Maria Lönn, Katrin Häggström Westberg, Ann‐Caroline Holst, and Lena‐Karin Erlandsson.

## Funding

This scoping review was written as part of a larger project, *Retrospective Evaluation of Occupational Therapy Group Interventions in Psychiatric Care*, which received funding from Sparbanksstiftelsen Region Halland (Grant Number 1011340).

## Conflicts of Interest

The authors declare no conflicts of interest.

## Supporting information


**Supporting Information** Additional supporting information can be found online in the Supporting Information section. References of included instruments are outlined in Supporting Information 1.

## Data Availability

The data supporting the findings of this study are available from the corresponding author upon reasonable request.
