# Supplementary material for: Occupational Therapy Group Interventions Within the Area of Time Use and Occupational Balance: A Scoping Review
Source: Occup Ther Int. 2026 Jun 25;2026:9972405. doi: 10.1155/oti/9972405 (PMC13305142; doi:10.1155/oti/9972405)
Supplement: Supplementary file 1 — Supporting Information Additional supporting information can be found online in the Supporting Information section. References of included instruments are outlined in Supporting Information 1. [file OTI-2026-9972405-s001.pdf]

SUPPLEMENTARY 1. References for the instruments included in the scoping review.

| Instrument | Reference |
|---|---|
| General Self-Efficacy Scale (GSE-10) | Schwarzer R, Jerusalem M. In: Weinman J, Wright S, Johnston M, editors. Generalized self-efficacy scale, in Measures in health psychology: a user's portfolio. Causal and control beliefs. Windsor, UK: NFER-NELSON; 1995. |
| University of Washington Self-Efficacy Scale (UWSES) | Amtmann D, Bamer AM, Cook KF, Askew RL, Noonan VK, Brockway JA. University of Washington self-efficacy scale: a new self-efficacy scale for people with disabilities. Arch Phys Med Rehabil. 2012 Oct;93(10):1757-65. doi: 10.1016/j.apmr.2012.05.001. Epub 2012 May 7. |
| Rosenberg Self-Esteem Scale (RSES) | Rosenberg M. Society and the adolescent self image. Princeton, NJ: Princeton University Press; 1965. |
| Revised Implicit Theories of Intelligence (Self-Theory) Scale (RITIS) | De Castella, K., Byrne, D. My intelligence may be more malleable than yours: the revised implicit theories of intelligence (self-theory) scale is a better predictor of achievement, motivation, and student disengagement. Eur J Psychol Educ 30, 245–267 (2015). doi: 10.1007/s10212-015-0244-y |
| Self-Compassion Scale – Short Form (SCS-SF) | Raes F, Pommier E, Neff KD, Van Gucht D. Construction and factorial validation of a short form of the Self-Compassion Scale. Clin Psychol Psychother. 2011 May-Jun;18(3):250-5. doi: 10.1002/cpp.702. Epub 2010 Jun 8. |
| The Pain Catastrophizing Scale (PCS) | Sullivan MJL, Bishop SR, Pivik J. The pain catastrophizing scale: development and validation. Psychol Assess 1995;7:524–32.<br>Osman A, Barrios FX, Kopper BA, Hauptmann W, Jones J, O'Neill E. Factor structure, reliability, and validity of the pain catastrophizing scale. J Behav Med 1997;20:589–605. |
| The Chronic Pain Acceptance Questionnaire (CPAQ-20 and CPAQ-8) | Eide H, Leren L, Sørebø Ø. The Norwegian versions of the Chronic Pain Acceptance Questionnaire CPAQ-20 and CPAQ-8: validation and reliability studies. Disabil Rehabil 2017;39:1441–8.<br>Rovner GS, Arestedt K, Gerdle B, Börsbo B, McCracken LM. Psychometric properties of the 8-item chronic pain acceptance questionnaire (CPAQ-8) in a Swedish chronic pain cohort. J Rehabil Med 2014;46:73–80.<br>Fish RA, McGuire B, Hogan M, Morrison TG, Stewart I. Validation of the chronic pain acceptance questionnaire (CPAQ) in an internet sample and development and preliminary validation of the CPAQ-8. Pain 2010; 149:435–43. |
| The Pain Self- Efficacy Questionnaire | Rasmussen MU, Rydahl-Hansen S, Amris K, Danneskiold Samsøe B, Mortensen EL. The adaptation of a Danish version of the Pain Self-Efficacy Questionnaire: reliability and construct validity in a population of patients with fibromyalgia in Denmark. Scand J Caring Sci 2016;30:202–10. |
| Pearlin Mastery Scale for Self-Mastery (PMSSM) | Pearlin L, Lieberman MA, Menaghan E, Mullan JT. The stress process. J Health Soc Behav. 1981;22:337–56. |
| Self-Efficacy for Performing Energy Conservation Strategies Assessment (SEPECSA) | Liepold A, Mathiowetz V. Reliability and validity of the Self-Efficacy for Performing Energy Conservation Strategies Assessment for persons with multiple sclerosis. Occup Ther Int. 2005;12(4):234-49. doi: 10.1002/oti.5. |
| Questionnaire about the Process of Recovery (QPR) | Argentzell E, Hultqvist J, Neil S, Eklund M. Measuring personal recovery - psychometric properties of the Swedish questionnaire about the process of recovery (QPR-Swe). Nord J Psychiatry. 2017;71(7):529–35 |
| Recovery Assessment Scale—Domains and Stages (RAS-DS) | Hancock, N.; Scanlan, J.N.; Bundy, A.C.; Honey, A. Recovery Assessment Scale–Domain & Stages (RAS-DS) Manual; University of Sydney: Sydney, Australia, 2014.<br>Scanlan, J.N.; Hancock, N.; Honey, A. The Recovery Assessment Scale-Domains and Stages (RAS-DS): Sensitivity to change over time and convergent validity with level of unmet need. Psychiatry Res. 2018, 216, 560–564. |
| Motivation (VAS) | Eklund M, Tjornstrand C. Psychiatric rehabilitation in community-based day centres: motivation and satisfaction. Scand J Occup Ther. 2013;20:438–445. |
| Desire to Drug Questionnaire (DDQ) | Hassani-Abharian P, Mokri A, Ganjgahi H, Oghabian MA, Ekhtiari H. Validation for Persian Versions of "Desire for Drug Questionnaire" and "Obsessive Compulsive Drug Use Scale" in Heroin Dependents. Arch Iran Med. 2016 Sep;19(9):659-65. |
| Canadian Occupational Performance Measure (COPM) | Law M, Baptiste S, McColl M, Opzoomer A, Polatajko H, Pollock N. The Canadian occupational performance measure: an outcome measure for |

| | |
|---|---|
| | occupational therapy. Can J Occup Ther. 1990 Apr;57(2):82-7. doi: 10.1177/000841749005700207. |
| Satisfaction with Daily Occupations and Occupational Balance (SDO-OB) | Eklund M, Argentzell E. Perception of occupational balance by people with mental illness: a new methodology. Scand J Occup Ther. 2016;23(4):304–13. |
| Satisfaction with Daily Occupations (SDO-13) | Eklund, M., Bäckström, M., & Eakman, A. M. (2014). Psychometric properties and factor structure of the 13-item Satisfaction With Daily Occupations scale when used with people with mental health problems. Health and Quality of Life Outcomes, 12, 191. doi: 10.1186/s12955-014-0191-3 |
| Occupational Self-Assessment – Short Form (OSA-SF) | Popova ES, Ostrowski RK, Wescott JJ, Taylor RR. Development and Validation of the Occupational Self-Assessment-Short Form (OSA-SF). Am J Occup Ther. 2019 May/Jun;73(3):7303205020p1-7303205020p10. doi: 10.5014/ajot.2019.030288. |
| Occupational Self-Assessment (OSA) | Kielhofner G, Dobria L, Forsyth K, Kramer J. The Occupational Self Assessment: Stability and the Ability to Detect Change over Time. OTJR: Occupational Therapy Journal of Research. 2010;30(1):11-19. doi:10.3928/15394492-20091214-03 |
| Occupational Questionnaire (OQ) | Smith NR, Kielhofner G, Watts JH. The relationships between volition, activity pattern, and life satisfaction in the elderly. Am J Occup Ther. 1986 Apr;40(4):278-83. doi: 10.5014/ajot.40.4.278. PMID: 3963137. |
| Hospital Anxiety and Depression Scale (HADS) | Zigmond, A. S. & Snaith, R. P. (1983). The hospital anxiety and depression scale. Acta Psychiatrica Scandinavica, 67, 361–370. |
| General Anxiety Disorder (GAD) | Spitzer, R. L., Kroenke, K., Williams, J. B. W., & Lowe, B. (2006). A brief measure for assessing generalized anxiety disorder: The GAD-7. Archives of Internal Medicine, 166(10), 1092–1097. doi: 10.1001/archinte.166.10.1092 |
| Depression Anxiety Stress Scale (DASS) | Lovibond, S. H., & Lovibond, P. F. (1995). Manual for the Depression Anxiety Stress Scales. Sydney: The Psychology Foundation of Australia. |
| The Perseived Stress Scale (PSS) | Cohen, S.; Kamarck, T.; & Mermelstein, R. (1983) A global measure of perceived stress. Journal of Health and Social Behavior, 24(4), 385. |
| Stress Response Inventory (SRI) | Koh KB, Park JK, Kim CH, Cho S. Development of the stress response inventory and its application in clinical practice. Psychosom Med. 2001 Jul-Aug;63(4):668-78. doi: 10.1097/00006842-200107000-00020. |
| The Modified Fatigue Impact Scale (MFIS) | Kos D, Duportail M, Meirte J, Meeus M, D'hooghe MB, Nagels G, Willekens B, Meurrens T, Ilsbroukx S, Nijs J. The effectiveness of a self-management occupational therapy intervention on activity performance in individuals with multiple sclerosis-related fatigue: a randomized-controlled trial. Int J Rehabil Res. 2016 Sep;39(3):255-62. doi: 10.1097/MRR.0000000000000178. |
| The Brief Pain Inventory (BPI)Pain intensity | Cleeland C, Ryan K. Pain assessment: global use of the brief pain inventory. Ann Acad Med Singapore 1994;23:129–38. |
| Body Pain Diagrams (BPD)(Pain spreading) | Southerst D, Cote P, Stupar M, Stern P, Mior S. The reliability of body pain diagrams in the quantitative measurement of pain distribution and location in patients with musculoskeletal pain: a systematic review. J Manip Physiol Ther 2013;36:450–9. |
| Behavior and Symptom Identification Scale (BASIS-24) | Cameron, I.; Cunningham, L.; Crawford, J.; Eagles, J.; Eisen, S.; Lawton, K.; Naji, S.; Hamilton, R. Psychometric properties of the BASIS-24 (c) (Behaviour and Symptom Identification Scale-revised) Mental Health Outcome Measure. Int. J. Psychiatry Clin. Pract. 2007, 11, 36–43. |
| Profiles of Occupational Engagement among people with Severe mental illness (POES) | Bejerholm U, Hansson L, Eklund M. Profiles of occupational engagement among people with schizophrenia: instrument development, content validity, inter-rater reliability, and internal consistency. Br J Occup Ther. 2006;69(2):58–68.<br>Bejerholm U, Lundgren-Nilsson A. Rasch analysis of the profiles of occupational engagement in people with severe mental illness (POES) instrument. Health Quol Life Outc. 2015;13:130. |
| The Engagement in Meaningful Activities Survey (EMAS) | Eakman AM. Measurement characteristics of the engagement in meaningful activities survey in an age-diverse sample. Am J Occup Ther. 2012 Mar-Apr;66(2):e20-9. doi: 10.5014/ajot.2012.001867. PMID: 22394537; PMCID: PMC3298038.<br>Goldberg, B., Brintnell, E. S., & Goldberg, J. (2002). The Relationship Between Engagement in Meaningful Activities and Quality of Life in Persons Disabled by Mental Illness. Occupational Therapy in Mental Health, 18(2), 17–44. doi:10.1300/J004v18n02_03 |
| Occupational Value (Oval-pd) | Eklund M, Erlandsson LK, Persson D. Occupational value |

| | |
|---|---|
| | among individuals with long-term mental illness. Can J Occup Ther 2003;70(5):276-84. Eklund M, Erlandsson LK, Persson D, Hagell P. Rasch analysis of an instrument for measuring occupational value: Implications for theory and practice. Scand J Occup Ther 2009;16(2):118-28. |
| Living in the Community Questionnaire (LCQ) | Australian Health Ministers Advisory Council Mental Health Information Strategy Standing Committee, Living in the Community Questionnaire (LCQ)—A Measure of Social Participaition: A Guide for the Use of the LCQ in Clinical Practice and for Service Development; National Mental Health Strategy: Canberra, Australia, 2016. |
| Nottingham Leisure Questionnaire (NLQ) | Drummond AE, Parker CJ, Gladman JR, Logan PA; TOTAL Study Group. Development and validation of the Nottingham Leisure Questionnaire (NLQ). Clin Rehabil. 2001 Dec;15(6):647-56. doi: 10.1191/0269215501cr438oa. PMID: 11777095. Altintas E, Guerrien A, Vivicorsi B, Clément E, Vallerand RJ. Leisure Activities and Motivational Profiles in Adaptation to Nursing Homes. Can J Aging. 2018 Sep;37(3):333-344. doi: 10.1017/S0714980818000156. Epub 2018 Jul 13. |
| Camberwell Assessment of Need Short Appraisal (CANSAS) | Slade, M.; Phelan, M.; Thornicroft, G.; Parkman, S. The Camberwell Assessment of Need (CAN): Comparison of assessments by staff and patients of the needs of the severely mentally ill. Soc. Psychiatry Psychiatr. Epidemiol. 1996, 31, 109–113. Trauer, T.; Tobias, G.; Slade, M. Development and evaluation of a patient-rated version of the Camberwell Assessment of Need Short Appraisal Schedule (CANSAS-P). Community Ment. Health J. 2008, 44, 113–124. |
| Global Assessment of Functioning (GAF) | Endicott J, Spitzer RL, Fleiss JL, Cohen J. The global assessment scale. A procedure for measuring overall severity of psychiatric disturbance. Arch Gen Psychiatry. 1976;33(6):766–71. |
| Worker Role Self-assessment (WRS) | Eklund M, Backström M. Factor structure and construct ¨ validity of the Worker Role Self-assessment (WRS) when used for people with psychiatric disabilities in Sweden. Eval Health Prof. 2016;39(3):299-316. Wastberg BA, Haglund L, Eklund M. Psychometric prop- erties of the Worker Role Self-assessment instrument used to evaluate unemployed people in Sweden. Scand J Occup Ther. 2009;16(4):238-46 |
| Work Ability Index (WAI) | Ahlstrom L, Grimby-Ekman A, Hagberg M, et al. The work ability index and single-item question: associations with sick leave, symptoms, and health–a prospective study of women on long-term sick leave. Scand J Work Environ Health. 2010;36:404–412. |
| 24 h time diary | Phipps, P.A.; Vernon, M.K. Twenty-Four Hours: An Overview of the Recall Diary Method and Data Quality in the American Time Use Survey. In Calendar and Time Diary: Methods in Life Course Research; Belli, R.F., Stafford, F.P., Alwin, D.F., Eds.; SAGE: Thousand Oaks, CA, USA, 2009. |
| Time of sick | Percentage of time off sick calculated as the proportion of the woman's normal working hours. |
| Sleep patterns (actigraphy) | Ameen, C., & Hauser, H. (2019). About the accuracy and problems of consumer devices in the assessment of sleep. Sensors, 19(19), 4160. https://doi.org/10.3390/s19194160 |
| RU-SATED Questionnaire | Ravyts SG, Dzierzewski JM, Perez EN, Donovan EK, Dautovich ND (2021). Sleep health as measured by RU SATED: a psychometric evaluation. Behavioral Sleep Medicine 19, 48–56 |
| Sleep quality (KSQ) | Nordin M, Åkerstedt T, Nordin S. Psychometric evaluation and normative data for the Karolinska sleep questionnaire. Sleep Biol Rhythms 2013;11:216–26 |
| Pittsburgh Sleep Quality Index (PSQI) | Backhaus, J., Junghanns, K., Broocks, A., Riemann, D., & Hohagen, F. (2002). Test-retest reliability and validity of the Pittsburgh sleep quality Index in primary insomnia. Journal of Psychosomatic Research, 53(3), 737–740.doi:10.1016/S0022-3999(02)00330-6 |
| Insomnia severity index (ISI) | Baghyahi, S. B. A., Zhang, X., Baghsiahi, H. R. B. A., Torabi, S., & Aval, M. B. (2011). Reliability and validity of the Chinese translation of Insomnia Severity Index (C-ISI) IN Chinese patients with insomnia. Sleep Medicine, 12(12) |

| | |
|---|---|
| | Bastien CH, Vallières A, Morin CM. Validation of the Insomnia Severity Index as an outcome measure for insomnia research. Sleep Med. 2001 Jul;2(4):297-307. doi: 10.1016/s1389-9457(00)00065-4. |
| Assessment of Time Management Skills (ATMS-S) | Janeslätt GK, Holmqvist KL, White S, Holmefur M. Assessment of time management skills: psychometric properties of the Swedish version. Scand J Occup Ther. 2018 May;25(3):153-161. doi: 10.1080/11038128.2017.1375009. |
| Weekly Calendar Planning Activity (WCPA) | Toglia J. Weekly Calendar Planning Activity (WCPA): a performance test of executive function. Bethesda, MD: AOTA Press, The American Occupational Therapy Association Inc.; 2015.<br>Holmqvist, KL, Holmefur, M, & Arvidsson, P. (2019). Test–retest reliability of the Swedish version of the Weekly Calendar Planning Activity – a performance-based test of executive functioning. Disabil Rehabil, 42(18), 2647–2652. Doi: 10.1080/09638288.2019.1568590. |
| Warwick-Edinburgh Mental Well-being Scale (WEMWBS) | Tennant R, Hiller L, Fishwick R, Platt S, Joseph S, Weich S, Parkinson J, Secker J, Stewart-Brown S. The Warwick-Edinburgh Mental Well-being Scale (WEMWBS): development and UK validation. Health Qual Life Outcomes. 2007 Nov 27;5:63. doi: 10.1186/1477-7525-5-63. |
| 14-item Scales of General Well-being (14-SGWB) | Ylenio Longo, Iain Coyne, Stephen Joseph, Development of the short version of the Scales of General Well-Being: The 14-item SGWB, Personality and Individual Differences, Volume 124, 2018, Pages 31-34, ISSN 0191-8869, doi:10.1016/j.paid.2017.11.042. |
| The Gratitude Questionnaire-6 (GQ-6) | Mccullough ME, Emmons RA, Tsang JA. The grateful disposition: a conceptual and empirical topography. J Pers Soc Psychol. 2002 Jan;82(1):112-27. doi: 10.1037//0022-3514.82.1.112. |
| 36-Item Short-Form Health Survey (SF-36) | Ware JE Jr, Sherbourne CD. The MOS 36-item short-form health survey (SF-36). I. Conceptual framework and item selection. Med Care. 1992 Jun;30(6):473-83. |
| The first item of the MOS SF-36 | Ware JE, Jr., Sherbourne CD: The MOS 36-item short-form health survey (SF-36). Conceptual framework and item selection. Med Care 1992, 30(6):473–483.<br>Bowling A. Just one question: if one question works, why ask several? J Epidemiol Community Health. 2005;59(5):342–5 |
| EQ5D | Devlin N, Parkin D, Janssen B. Methods for analysing and reporting EQ-5D data, 2020. Cham (CH): Springer. Available: https://www.ncbi.nlm.nih.gov/books/NBK565678/<br>Janssen MF, Pickard AS, Golicki D, et al. Measurement properties of the EQ-5D-5L compared to the EQ-5D-3L across eight patient groups: a multi-country study. Qual Life Res 2013;22:1717–27.<br>Herdman M, Gudex C, Lloyd A, et al. Development and preliminary testing of the new five-level version of EQ-5D (EQ-5D-5L). Qual Life Res 2011;20:1727–36. |
| Personal Health Questionnaire 9 (PHQ9) | Löwe B, Unützer J, Callahan CM, Perkins AJ, Kroenke K. Monitoring depression treatment outcomes with the patient health questionnaire-9. Med Care. 2004 Dec;42(12):1194-201. doi: 10.1097/00005650-200412000-00006. |
| Health and disability (WHODAS) | editors, T.B̈. Üstün ... [and others]. (2010). Measuring health and disability : manual for WHO Disability Assessment Schedule WHODAS 2.0. Geneva :World Health Organization. |
| Occupational Balance Questionnaire (OBQ11) | Håkansson C, Wagman P, Hagell P. Construct validity of a revised version of the Occupational Balance Questionnaire. Scand J Occup Ther. 2020 Aug;27(6):441-449. doi: 10.1080/11038128.2019.1660801. Epub 2019 Sep 14. |
| Ocupational Balance Questionnaire (OBQ) | Wagman P, Håkansson C. Introducing the occupational balance questionnaire (OBQ). Article. Scand J Occup Ther 2014;21:227–31. |
| Ocupational Balance Questionnaire (OB-Quest) | Dür, M., Steiner, G., Fialka-Moser, V., Kautzky-Willer, A., Dejaco, C., Prodinger, B., Stoffer, M. A., Binder, A., Smolen, J., & Stamm, T. A. (2014). Development of a new occupational balance-questionnaire: Incorporating the perspectives of patients and healthy people in the design of a self-reported occupational balance outcome instrument. Health and Quality of Life Outcomes, 12(1), 1–11. doi: 10.1186/1477-7525-12-45 |

| | |
|---|---|
| Manchester Short Assessment of Quality of Life (MANSA) | Priebe S, Huxley P, Knight S, Evans S. Application and results of the Manchester short assessment of quality of life (MANSA). Int J Soc Psychiatry. 1999;45(1):7–12. |
| World Health Organization Quality of Life Scale Abbreviated Version (WHOQOL-BREF) | Min SK, Kim KI, Lee CI, Jung YC, Suh SY, Kim DK. Development of the Korean versions of WHO Quality of Life scale and WHOQOL-BREF. Qual Life Res. 2002 Sep;11(6):593-600. doi: 10.1023/a:1016351406336. |
| The Work Environment Impact Scale – Self-Rating(WEIS-SR) | Wastberg, B. A., Haglund, L. & Eklund, M. (2012). The Work Environment Impact Scale-Self-Rating (WEIS-SR) evaluated in primary health care in Sweden. Work, 42, 1–12. |
| Interview Schedule for Social Integration (ISSI-SR) | Unden AL, Orth-Gomer K. Development of a social support instrument for use in population surveys. Soc Sci Med.1989;29(12):1387-92.<br>Eklund M, Bengtsson-Tops A, Lindstedt H. Construct and discriminant validity and dimensionality of the Interview Schedule for Social Interaction (ISSI) in three psychiatric samples. Nord J Psychiatry. 2007;61(3):182-8. |
